# Tackling Chronic Inflammation with Withanolide Phytochemicals—A Withaferin A Perspective

**DOI:** 10.3390/antiox9111107

**Published:** 2020-11-10

**Authors:** Emilie Logie, Wim Vanden Berghe

**Affiliations:** Lab Protein Chemistry, Proteomics & Epigenetic Signaling (PPES), Department of Biomedical Sciences, University of Antwerp, 2610 Wilrijk, Belgium; wim.vandenberghe@uantwerpen.be

**Keywords:** Withaferin A, natural product, inflammation, chronic diseases, therapy

## Abstract

Chronic inflammatory diseases are considered to be one of the biggest threats to human health. Most prescribed pharmaceutical drugs aiming to treat these diseases are characterized by side-effects and negatively affect therapy adherence. Finding alternative treatment strategies to tackle chronic inflammation has therefore been gaining interest over the last few decades. In this context, Withaferin A (WA), a natural bioactive compound isolated from *Withania somnifera*, has been identified as a promising anti-cancer and anti-inflammatory compound. Although the majority of studies focus on the molecular mechanisms of WA in cancer models, recent evidence demonstrates that WA also holds promise as a new phytotherapeutic agent against chronic inflammatory diseases. By targeting crucial inflammatory pathways, including nuclear factor kappa B (NF-κB) and nuclear factor erythroid 2 related factor 2 (Nrf2) signaling, WA suppresses the inflammatory disease state in several in vitro and preclinical in vivo models of diabetes, obesity, neurodegenerative disorders, cystic fibrosis and osteoarthritis. This review provides a concise overview of the molecular mechanisms by which WA orchestrates its anti-inflammatory effects to restore immune homeostasis.

## 1. Introduction

Inflammation is a complex biological response to harmful triggers such as infection, tissue injury or tissue stress [1]. In healthy individuals, this inflammatory defense is tightly controlled by a plethora of inhibitory feedback mechanisms orchestrated by immune cells in order to minimize exacerbated tissue damage [2,3,4,5,6,7]. However, when the inflammatory stimulus is persistent, a chronic immune response can be prompted, leading to a prolonged increase of pro-inflammatory mediators [8]. Depending on the duration and severity of the chronic inflammation, irreversible tissue damage and/or fibrogenesis might be initiated, leading to a variety of chronic diseases including cardiovascular diseases, diabetes type 2, rheumatoid arthritis, degenerative brain diseases and cancer [9,10,11,12,13,14,15].

As the World Health Organization (WHO) ranks chronic non-communicable diseases as one of the major threats to human health [16], researchers are focusing on finding effective treatment and prevention strategies to cure chronic inflammation and its consequences. Currently, the most prescribed pharmacological drugs to treat chronic diseases include metformin, statins, non-steroidal anti-inflammatory drugs (NSAIDs) and corticosteroids [17,18]. Although these compounds have proven their efficacy in the clinical setting, most of them are characterized by side-effects which negatively affect treatment adherence [19]. Frequently reported adverse effects of corticosteroids, for example, include osteoporosis, adrenal suppression, hyperglycemia, dyslipidemia, Cushing’s syndrome, psychiatric disturbances and immunosuppression [20]. Thus, in order to improve patient quality of life using these commonly prescribed drugs, there is a growing interest in finding alternative or supplemental treatment strategies to restore immune homeostasis.

Knowing that many Food and Drug Administration (FDA)-approved drugs are natural products and derivatives, the extensive arsenal of plant compounds is currently being explored and exploited for the treatment of chronic diseases [21,22,23,24]. Used for millennia in traditional ethnomedicine, herbal treatments are a promising alternative to existing therapies, with lower rates of adverse events and efficiency frequently comparable to that of conventional drugs. In an attempt to identify novel anti-inflammatory agents which are safe and effective, a “reverse pharmacology” or “bed to bench-side” approach is applied by bioactivity-guided fractionation of plants which have been used for centuries to reduce inflammatory symptoms [25]. Bioactive phytochemicals from ginger (*Zingiber officinale*) or turmeric (*Curcuma longa*), for example, have already shown therapeutic efficacy in patients suffering from inflammatory conditions such as osteoarthritis [26,27]. The main bioactive constituent of the *Corida verbenacea* plant has even been commercialized into the anti-inflammatory phytotherapeutic agent Acheflan^®^ and is used as a topical cream to treat local inflammation [28]. Another popular traditional ethnomedicinal herb displaying several anti-inflammatory properties is *Withania somnifera*, also known as Ashwagandha or Indian Winter Cherry. Roots and berries from this plant have been used for over 3000 years in Ayurvedic medicine and are described to remedy chronic fatigue, dehydration, rheumatism an ulcers [29]. Here, we will summarize the polypharmacological mechanisms of action of one of its most promising bioactive constituents, Withaferin A, which supports resolution of various chronic diseases.

## 2. Withaferin A: The Major Bioactive Constituent Isolated from *Withania somnifera*

**Figure 1:**
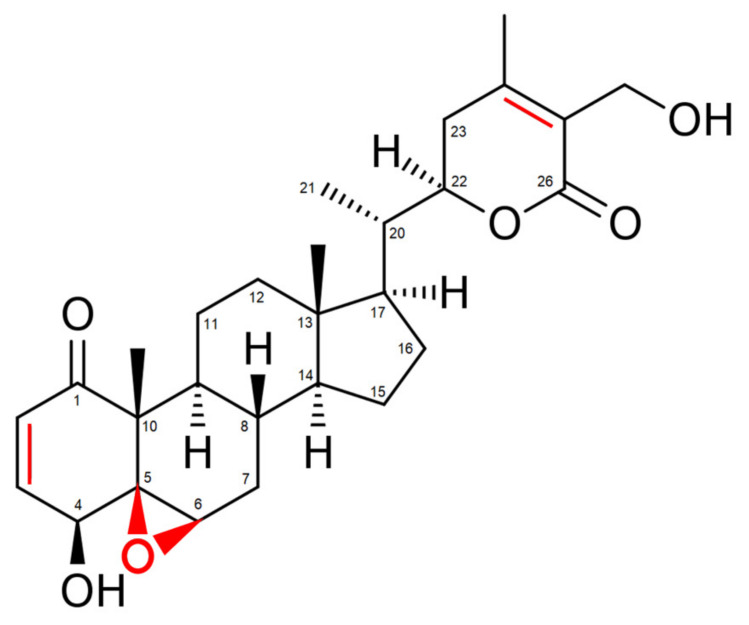
Structure of Withaferin A (WA). Regions prone to nucleophilic attacks are marked in red.

*Withania somnifera* comprises over 35 chemical constituents of which the alkaloids, flavonoids, steroidal lactones and saponins are biologically active [30]. However, the most potent bioactive compound isolated from *Withania somnifera* roots is the highly oxygenated lactone Withaferin A (WA). Indeed, studies show that most beneficial health effects of *Withania somnifera*, ranging from anti-inflammatory to anti-cancer effects, can be attributed to WA [31,32,33,34]. These effects are mostly accomplished via the covalent binding of WA with target proteins, resulting in a loss of activity of the latter [29]. Three sites in particular, namely the unsaturated A-ring at C3, the epoxide structure at position 5 and C24 in its E-ring, are especially prone to nucleophilic attacks and are often involved in Michael addition alkylation reactions [29] (Figure 1).

## 3. Molecular Targets of Withaferin A in the Inflammatory Response Pathway

**Figure 2:**
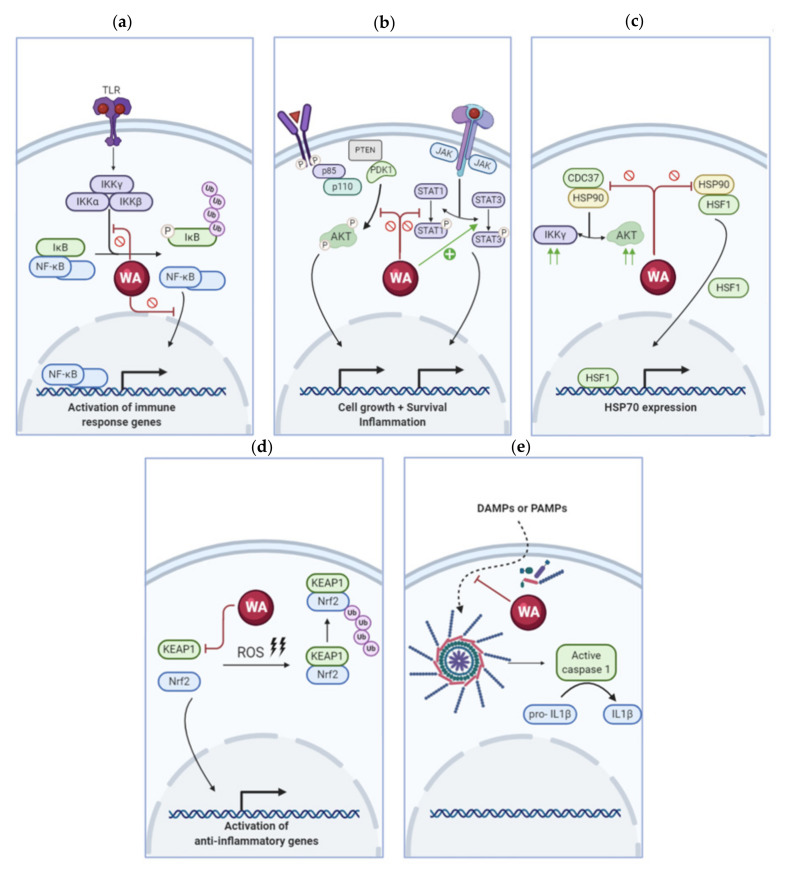
Overview of inflammatory signaling pathways altered by WA. (**a**) WA inhibits NF-κB signaling; (**b**) Several kinase signaling pathways, such as AKT/mTOR and JAK/STAT, are regulated by WA; (**c**) WA mediates the heat shock response by inhibiting HSP90; (**d**) Expression of anti-inflammatory genes is promoted by WA through activation of Nrf2 signaling; (**e**) WA prevents NLRP3 inflammasome formation and activation. Abbreviations: TLR, Toll-like receptor; IKK, IκB kinase; NF-κB, Nuclear Factor kappa B; WA, Withaferin A; JAK, Janus kinase; STAT, Signal Transducers and Activators of Transcription; PTEN, Phosphatase and Tensin Homolog; PDK1, Phosphoinositide-dependent kinase-1; HSP90, Heat Shock Protein 90; HSF1, Heat Shock Factor 1; CDC37, Cell Division Cycle 37; KEAP1, Kelch-like ECH-associated Protein; Nrf2, Nuclear Factor Erythroid 2-related Factor; ROS, Reactive Oxygen Species; DAMP, Damage-associated Molecular Pattern; PAMP, Pathogen-associated Molecular Pattern; IL1β, Interleukin-1β.

The anti-inflammatory activities of WA have already been demonstrated in a wide variety of chronic disease animal models (Table 1). Reduced inflammation and overall alleviation of disease symptoms have been documented in conditions such as cystic fibrosis, diabetes and pulmonary fibrosis [35,36,37]. Although its complete mechanism of action has not yet been fully resolved, WA interacts with several mediators of the inflammatory cell signaling pathway including NF-κB, signaling kinases (e.g., JAK/STAT), heat shock protein 90 (HSP90), Nrf2 and the inflammasome complex. A concise overview of the effects of WA on these pathways is illustrated in Figure 2.

### 3.1. Withaferin A Inhibits the NF-κB Pathway

The family of NF-κB transcription factors is one of the major orchestrators of the inflammatory immune response [38]. Through ligation of pro-inflammatory cytokines or pathogen-derived substances to their corresponding cognate receptors, NF-κB is rapidly activated and regulates the expression of growth factors, prostaglandins, cytokines, inducible nitric oxide synthase (iNOS) and cyclooxygenase-2 (COX-2) to promote cell growth, cell survival and angiogenesis [1,38]. Because of its importance in the regulation of cell survival and expression of inflammatory mediators, the activation of NF-κB is strictly regulated by its cytoplasmic inhibitor IκB. This inhibitor masks the nuclear localization signal of NF-κB, preventing nuclear translocation and subsequent activation of target genes [39]. Upon exposure to inflammatory stimuli however, the IκB inhibitor is phosphorylated by the IκB-kinase (IKK) complex, composed of a regulatory subunit IKKγ (known as NEMO) and two kinase subunits, IKKα and IKKβ, leading to proteasomal degradation and allowing activation of NF-κB responsive genes.

Unsurprisingly, many inflammation-driven chronic diseases are characterized by constitutively active NF-κB, making it an interesting therapeutic target [40]. In this regard, WA has been extensively studied in models of chronic inflammation to investigate its effect on NF-κB signaling. Molecular docking studies demonstrate that WA is indeed able to interfere with the NF-κB pathway through several mechanisms. One particular in silico analysis has demonstrated a strong possible intermolecular interaction between WA and IKKγ, which disrupts formation of the IKK complex and prevents IκB degradation [41]. Alternatively, in vitro IKK kinase assays have shown that WA directly interacts with IKKβ, rather than IKKγ, through targeting of cysteine 179 [42,43]. Reduction in IKK activation by WA could also be observed in different in vitro and in vivo models of chronic diseases, including lung fibrosis [30], obesity [44], scleroderma [45] and cancer [43]. Other suggested molecular mechanisms of WA-mediated inhibition of NF-κB are direct interaction of WA with NF-κB itself [46] or its IκB inhibitor [47]. The former mechanism has been proposed by Ashkenazi and colleagues [46] whose computational modeling suggests that WA interferes with p65 dimerization, which is crucial for DNA binding of NF-κB.

While it remains uncertain which of the mentioned mechanisms are pre-dominant, WA shows promise as a therapeutic intervention in diseases with chronically elevated levels of NF-κB levels such as Alzheimer [48], diabetes [36] and cystic fibrosis [35].

### 3.2. Modulation of Kinase Activity by Withaferin A

Protein kinases (PK) are involved in many cellular processes and an increasing number of studies demonstrate that inflammation is no exception. By catalyzing the transfer of phosphate groups to specific substrates, kinases are able to propagate intracellular signals and regulate the biological activity of their target. This phosphorylation signaling cascade is crucial in the inflammatory pathway where the initiation, propagation an regulation of immunological responses heavily rely on PK [49]. For this reason, the development of potent kinase inhibitors as a therapeutic strategy to treat chronic inflammation has gained a great deal of interest [50].

**Table 1:** Overview of animal models used to study the anti-inflammatory properties of Withaferin A.

Chronic Disease	Animal Model	Dose WA (mg/kg)	Notable Changes Observed	Reference(s)
**Amyotrophic lateral sclerosis**	TDP-43 transgenic C57BL/6J mice	3 mg/kg	Reduced inflammation Improved motor behavior	[51]
	SOD1 transgenic C57BL/6J mice	4 mg/kg	Extended survival Reduction of early neuronal injury response Reduced SOD1 misfolding Reduced neuroinflammatory signals	[52]
**Gliosis**	Excitotoxic model of inner retinal injury in C57BL/6J mice	2 mg/kg	Reduced inner retinal apoptosis Reduced levels of intermediate filaments	[53]
**Parkinson’s disease**	Age-mediated impairment of the dopamine system in Wistar Albino rats	50 mg/kg	Improved motor behavior Resurge of dopamine in substantia nigra and striatum	[54,55]
**Liver fibrosis**	Bile duct ligation induced liver fibrosis in C57BL/6J mice	1–3 mg/kg	Restoration of liver function and tissue architecture Reduction of collagen deposition Attenuated oxidative stress Reduced NF-κB expression	[44]
**Obesity**	High fat diet induced obesity in C57BL/6J mice	1.25 mg/kg	Reduced obesity-associated abnormalities (hepatic steatosis) Increased leptin potency Improved hepatic insulin sensitivity Enhanced glucose tolerance and glucose homeostasis Reduction in pro-inflammatory cytokines Enhanced lipid and glucose metabolism	[56,57]
	High fat diet induced obesity in C57BL/6J mice	10 mg/kg	Reduced weight gain Lower epididymal and mesenteric fat pad mass Improved lipid profile Reduction in inflammatory cytokines	[58]
**Diabetes**	Streptozotocin induced diabetes in Swiss albino mice	10 mg/kg	Reduced diabetes incidence Reduced hyperglycemia Improved glucose clearance	[36]
**Arthritis**	Monosodium urate crystal- induced inflammation as Gouty arthritis model	30 mg/kg	Reduced paw volume Reduced lipid peroxidation Reduced TNF-α levels Reduced levels of β-glucuronidase and lactate dehydrogenase	[59]
	Intradermal induction of rheumatoid arthritis in Albino Wistar rats	30 mg/kg free WA or 10 mg/kg liposomal WA	Reduced oxidative stress Reduced paw edema No bone erosion or cartilage degradation Increase in anti-inflammatory cytokines (IL-10)	[60]

Interestingly, WA targets several PK pivotal in inflammation such as AKT/mTOR [61], MAPK [62] and JAK/STAT [63] signaling pathways, other than IKK [42]. In a study by Mehta et al. [61], pretreatment of donor grafts with low doses of WA (1 µM) entirely blocked the post-transplant cytokine storm associated with graft-vs-host-diseases, significantly improving engraftment in mice transplanted with WA-treated bone marrow cells. To further explain the prophylactic action of WA, the authors investigated the levels of phosphorylated AKT in WA-treated splenocytes as the AKT/mTOR pathway is important in T-cell activation and proliferation. Surprisingly, complete inhibition of AKT phosphorylation was observed in addition to reduced levels of PDK1- and S6 kinases phosphorylation, upstream and downstream players of the AKT/mTOR pathway respectively, indicating that WA is able to block AKT signaling at multiple levels. Similar effects on AKT phosphorylation have also been observed in in vivo models of airway inflammation [64].

WA has also been demonstrated to inhibit JAK/STAT signaling, crucial for the transduction of signals arising from cytokine and growth factor receptors [65]. In BV-2 and primary microglial cells, treatment with WA prevented STAT1 phosphorylation and nuclear translocation, thereby inhibiting lipopolysacharide (LPS)-induced COX-2 and prostaglandin E2 (PGE_2_) expression [63]. Given that hyperactivated microglial cells are often related to the initiation of neurodegenerative diseases such as Alzheimer’s disease or amyotrophic lateral sclerosis (ALS), modulation of their inflammatory response by WA could hold promise in the prevention of brain tissue damage [66,67]. In contrast, another study using RAW 264.7 M1 macrophages suggests WA induces its anti-inflammatory effects via promotion of STAT phosphorylation, rather than inhibition [68]. Here, internalization of WA-decorated liposomes into activated, pro-inflammatory M1 macrophages increased STAT3 phosphorylation and ablated oxidative stress markers, leading to the repolarization of these macrophages to the anti-inflammatory M2 macrophage subtype [68]. These contradictory observations might reflect that the effect of WA treatment is dependent on cell type, dosage or incubation time [29].

Because some chronic diseases are caused by persistent bacterial infections, the inhibitory effects of WA on PK in response to these pathogens have also been explored [33,62,69]. In case of periodontitis, a progressive chronic infection of the periodontal tissue caused by infection with Gram-negative bacteria, MAPK activity in activated macrophages is impaired by WA in a dose-dependent manner [62]. This inactivation of kinase inhibition occurs in parallel with inhibition of iNOS expression and nitric oxide (NO) production, effectively halting progressive inflammation. Surprisingly, many experimental (mainly cancer) models investigating the therapeutic properties of WA on MAPK signaling demonstrate an activating rather than an inhibitory effect [70,71,72]. This highlights again that the molecular mechanism of WA is highly dependent on cellular context.

### 3.3. Withaferin A Regulates Heat Shock Proteins

Although there are several examples demonstrating the interplay between WA and PK, it remains uncertain whether any of the observed changes on kinase level are a direct result of WA interaction or rather a secondary effect. A relevant illustration of the latter is the ability of WA to regulate the activity of heat shock proteins (HSP), which are highly conserved molecular chaperones involved in the folding, transport, maintenance and assembly of key regulatory proteins like kinases [73]. By targeting and dissociating the CDC37-HSP90 complex, either via blocking the protein cleft of CDC37 [74] or via direct binding of HSP90 itself [75], WA downregulates HSP90 target proteins, such as AKT and the IKK-complex. The therapeutic relevance of CDC37-HSP90 blockage by WA has already been demonstrated in N9 microglial cells, where one particular kinase HSP90 target, Leucine-Rich Repeat Kinase 2 (LRRK2), is significantly downregulated and destabilized in a dose- and time-dependent manner after WA exposure without severely affecting cell viability [76]. LRRK2 is a multi-domain protein with an unidentified function, which is often mutated in patients with familial and sporadic Parkinson’s disease, Alzheimer’s disease and Crohn’s disease. Most mutations in this protein kinase are associated with gain-of-function activities, including increased protein expression and/or elevated kinase activity, crucial in the inflammatory disease etiology [76,77,78].

In contrast to HSP90 inhibition, SILAC-based proteomics analysis of the same N9 microglial cell line treated with WA showed an increased HSP70 response [79]. Other studies performed in in vivo models of chronic diseases, such as ALS, report induction of certain HSP by WA as well, emphasizing the beneficial therapeutic effects of this upregulation [52]. Although the upstream mediators elucidating the HSP70 increase were not further investigated within these experiments, HSP90 inhibition by WA could offer a plausible explanation for this observation. The heat shock response is driven by heat shock factor protein 1 (HSF1), a stress-inducible transcription factor that is able to induce the expression of different HSP, including HSP70 [80]. Under homeostatic conditions, HSF1 is sequestered in the cytoplasm as an inactive monomer by a protein complex containing HSP90 [81]. In the presence of a cellular stressor (e.g., oxidative stress, nutrient deficiency, etc.), HSF1 is activated and shuttled to the nucleus, leading to an increased expression of HSP70 and other targets [80,82]. Given that WA is able to inhibit HSP90 activity, HSP70 upregulation might result from a WA-induced (HSF1-dependent) heat shock response [83].

### 3.4. Withaferin A Alters the Cellular Redox Balance through Nrf2 Regulation

Reactive oxygen (ROS) and nitrogen (RNS) species are generated as by-products of the cellular metabolism, with the electron transport chain and cytochrome P450 enzymes being the primary ROS sources [84]. ROS are also produced by effector cells of the immune system (e.g., macrophages) where they serve as signaling molecules to further progress the inflammatory response and activate crucial inflammatory pathways like NF-κB signaling and inflammasome formation [85]. In healthy humans, the production of ROS/RNS is tightly regulated by antioxidant defenses, as an uncontrolled ROS generation may cause potential damage to all biomolecules, resulting in functional impairment and cell death. When the balance shifts in favor of the oxidants and the antioxidant defense becomes exhausted, redox signaling is disrupted and the cell suffers from oxidative stress [84]. Sites of inflammation are often characterized by the occurrence of oxidative stress, as the subsequent induced tissue damage aims to eliminate the inflammatory trigger. In case of chronic inflammation, the persistent presence of oxidative stress often leads to excessive damage causing a plethora of health issues [86]. However, oxidative stress itself might spark the development of chronic inflammation [87]. To this day, the intricate relationship between oxidative stress and chronic inflammation is being further explored as this might offer new therapeutical strategies [88].

Several studies have shown that WA is able to modulate oxidative stress through different mechanisms, although the effect of WA on ROS levels is highly dependent on cellular context [89]. In cancer cells, where the basal ROS levels are already relatively high, WA is able to further increase oxidative stress and promote cell death [90,91]. This is in stark contrast with the chronic inflammatory disease setting, where WA attenuates oxidative stress and suppresses ROS production, thereby decreasing disease severity [56,60,92,93]. This suppression is often mediated by activation of the anti-inflammatory Nrf2 pathway, which inhibits several inflammatory mediators and enzymes and activates antioxidant target genes [94]. Through a direct interaction with Kelch-like ECH associated protein (KEAP1), a substrate adaptor for cullin-based E3 ubiquitin ligase which controls the half-life of Nrf2, WA prevents NRF2 ubiquitination and proteasomal degradation [95,96]. Even when basal levels of Nrf2 are low, as is often the case in chronic inflammation diseases, WA is able to restore Nrf2 expression and decrease oxidative stress [36,44,97]. Importantly, WA-mediated activation of Nrf2 is sometimes dependent on the stage of the disease as well. For example, when WA was administered to mice suffering from late-stage nonalcoholic steatohepatitis (NASH), mRNA levels of genes involved in the Nrf2 were decreased while a clear upregulation of Nrf2 expression was measured in early-stage NASH animals [98].

Alternatively, WA was found to inhibit oxidative stress through Sirtuin 3 (Sirt3) activation [99]. Sirt3 is a class III histone deacetylase enzyme that is predominantly located in the mitochondria, where it is responsible for regulating post-translational modifications of target proteins and finetuning their activity. Since more than 65% of mitochondrial proteins are acetylated, Sirt3 plays a vital role in mitochondrial function and ROS production [100]. Through deacetylation of key proteins (e.g., Ku70 & HIF1a), mitochondrial metabolism, oxidative stress, cell survival and, —longevity are all regulated by Sirt3. Indeed, knockout of Sirt3 expression in mice resulted in a significant increase in ROS levels and a decrease in ATP production [101,102]. Given that Sirt3 is involved in maintaining the cellular redox balance by quenching ROS, it is not surprising that Sirt3 dysregulation has been linked to development of inflammatory organ injury [103]. Upregulation of Sirt3 expression might therefore be beneficial in the treatment of chronic inflammation [104,105]. In this context, WA was able to prevent PDGF-BB- and CCl_4_^−^ liver fibrosis through upregulation of Sirt3 [99]. Remarkably, the antifibrotic and antioxidant effect of WA was attenuated in Sirt3^−/−^ KO mice, suggesting the anti-inflammatory effect of WA is highly dependent on Sirt3 expression levels.

Next to regulating Nrf2 and Sirt3 activity, low concentrations of WA (<1 µM) also stimulate the expression of several other antioxidant proteins including glutathione (GSH), glutathione peroxidase (GPX), glutathione S-transferase (GST), catalase (CAT) and superoxide dismutase (SOD) [45,106,107,108] and lowers oxidative stress markers such as malondialdehyde (MDA) [36,44]. The combinatorial effect of WA on GSH, GPX enzymes and MDA levels might be worth further investigation as their expression and activity are critical determinants of ferroptosis, an iron-dependent form of cell death [109]. As ferroptosis has recently been linked to (chronic) inflammation, inhibition of this mode of cell death by WA might prove to be beneficial [110]. In contrast, high doses of WA (>10 µM) were shown to promote ferroptotic cell death in therapy resistant cancer models [111].

### 3.5. Withaferin A Affects Inflammasome Activation

Many processes within the inflammatory response are dependent on the release of intercellular messengers, of which cytokines in particular are indispensable for the regulation of the immune system. Within the broad repertoire of the cytokine family, a small subset (e.g., pro-inflammatory cytokines IL-1β and IL-18) require cleavage into their bioactive form before they are released into the extracellular environment where they can interact with their target receptors. Interestingly, WA can interfere with this cleavage process and thus lower the levels of key pro-inflammatory cytokines by regulating inflammasome activity [64,112,113]. Inflammasomes are a group of multiprotein complexes that assemble within the cytosol after sensing pattern- or danger-associated molecular patterns (PAMPs or DAMPs respectively) [114]. Once assembled, they serve as a scaffold to recruit the inactive zymogen pro-caspase-1, which leads to oligomerization of pro-caspase-1 proteins, triggering their autoproteolytic cleavage into the biologically active caspase-1 enzyme. Through the subsequent cleavage of pro-IL-1β and pro-IL-18, caspase-1 enables the release of these proinflammatory cytokines into the extracellular environment. Currently, two major classes of inflammasomes have been identified based on the presence of their intracellular receptor: the Nod-like receptor (NLR) inflammasomes and the AIM2-like receptor (ALR) inflammasomes [115]. Several research groups using inflammatory disease models have demonstrated that WA interferes with the formation of NLRP3 inflammasomes [64,113,116,117], an important subtype of the NLR class. Activation of the NLRP3 inflammasome is generally induced by the presence of pathogens, toxins, crystals, protein aggregates such as β-amyloid and DAMPs [118]. In context of (chronic) inflammatory diseases however, abnormal NLRP3 activation has been reported to play a role in disease initiation and progression [119]. Although the underlying molecular mechanism of WA-mediated inhibition of NLRP3 inflammasome formation needs to be further explored, medicinal use of (dietary) WA might offer an opportunity to regulate cytokine activation in inflammation-associated diseases [116]. It is important to note that the effect of WA on other inflammasome subtypes needs to be investigated as well, since Ngoungoure and colleagues demonstrated that WA treatment of M2 macrophages increased expression and activation of AIM2 inflammasomes, resulting in a pro-inflammatory rather than an anti-inflammatory response in immune cells [112].

### 3.6. Other Inflammatory Factors Targeted by Withaferin A

Besides the listed mechanisms, the anti-inflammatory properties of WA could also be attributed to its interaction with the peroxisome proliferator-activated receptors (PPAR), mainly known for their role in the lipid metabolism. The PPAR transcription factors belong to the family of nuclear receptors and are characterized by their ability to regulate gene expression upon ligand binding [120]. Known to halt NF-κB inflammation through their inhibitory effect on NF-κB and activator protein-1 (AP-1) [121,122,123], the PPAR factors are currently being investigated as a therapeutic target in chronic diseases [124,125]. A number of research papers demonstrate that WA changes PPAR expression levels, although the directionality of the altered expression is conflicting. An in vitro study using 3T3-L1 adipocytes has demonstrated that WA downregulates PPAR through phosphorylation of MAPK [126], while an in vivo experiment investigating high fat diet induced obesity in mice showed that WA upregulates PPAR hepatic mRNA expression [56]. Given that upregulation of PPAR expression is beneficial to treat chronic inflammatory conditions, further in vivo research in different disease models is needed to determine WA’s exact role on PPAR expression during inflammation.

Finally, WA is known to disrupt the structural dynamics of type III intermediate filament proteins (IF), including vimentin, desmin and glial fibrillary acidic protein (GFAP). Though the link between IF organization and chronic inflammation remains unclear, some chronic inflammation disorders have been reported to have with increased expression of IF proteins [127,128]. This is especially true for neurodegenerative diseases where astrocyte reactivity is often associated with increased expression and polymerization of IF [53]. Affinity purification (using biotinylated WA) and LC-MS/MS analysis confirmed the interaction of WA with IF and even identified the WA binding sites of these proteins [129,130,131,132]. Through this covalent interaction with IF, WA perturbs structural organization of IF by lowering the amount of filaments and causing the formation of short aggregates [53,129,132]. The therapeutic implication of this molecular mechanism of WA has recently been exploited in an in vivo setup of reactive gliosis by Livne-Bar et al. [53]. By targeting the IF in this model, WA reduced astrocyte and Müller glial reactivity leading to a blockage of TNF-α mediated neuronal apoptosis [53]. Given that reactive gliosis is a common pathological trait of most neurodegenerative diseases, WA-moderated protection of neurons in vivo might hold promise in the therapy for patients suffering from these disorders.

## 4. Future Perspectives

Compared to most synthetic drugs, which are designed to have a high selectivity and few off-target effects, WA is a natural compound with the ability to target several proteins simultaneously. This multi-target approach might be beneficial in treating anti-inflammatory disease where an imbalance exists between multiple pro- and anti-inflammatory players [133,134,135]. However, the risk of adverse effects also significantly increases when multiple cellular proteins are targeted at once. To this day, no clinical studies focusing on the safety and pharmacokinetic profile of WA have been performed in chronic inflammation, highlighting the need for further research in this area. A recent phase I trial of WA in advanced stage high grade osteosarcoma patients did reveal WA was generally well tolerated [136], yet it remains difficult to extrapolate this observation to other disease types. Similarly, crucial pharmacokinetic parameters, including absorption, have mainly been evaluated in rodents [137,138,139]. The overall pharmacokinetic profile of WA in these models was shown to be favorable, although the oral bioavailability was sometimes lower due to substantial first-pass hepatic metabolism [139]. To overcome this potential bioavailability barrier, new formulations of WA are currently being investigated [111,140].

An alternative strategy to minimize potential adverse effects and lower the doses of WA could be to investigate the synergistic effects of WA with other anti-inflammatory drugs. This approach is already being broadly investigated in several cancer models [141,142]. In a clinical trial combining the use of five Ayurvedic herbal formulations (*Zingiber officinale*, *Tinospora cordifolia*, *Emblica officinalis*, *Tribulus terrestris* and *Withania somnifera*) in the treatment of osteoarthritis seemed to significantly relieve pain in the majority of patients [143]. Another trial examined the synergistic effect of *Withania somnifera* extracts with antipsychotic treatment in schizophrenia and found that the combination treatment alleviated overall symptoms and stress [144]. Although both studies reported a beneficial effect of *Withania somnifera* extract, the ameliorated effect on patient’s symptoms could be partially attributed to the mix of withanolides present in the extract as well. Unfortunately, no combination treatments with pure WA have been tested yet in clinical intervention trials.

## 5. Conclusions

Since its discovery in the late 1960s, WA has been extensively studied for its anti-cancer and anti-inflammatory effects. Although the majority of research focusses on the anti-cancer properties of WA (reviewed in References [89,145]), this review summarizes its therapeutic potential in chronic inflammatory diseases. The molecular pathways targeted by WA treatment in chronic inflammation are highly similar to those affected in cancer models, albeit the downstream effects on protein expression or activation sometimes differ depending on concentration and cell type context. By interacting with the NF-κB pathway, PK, heat shock proteins, Nrf2 signaling and inflammasome activation, WA decreases the inflammatory response in various in vitro and in vivo models. The therapeutic use of WA in a pre-clinical setting, however, has not yet been thoroughly investigated. Future research in humans is necessary to investigate whether the pharmacological and toxicological properties of WA allow for its implementation as an anti-inflammatory drug.
